# Evidence Linking *PPARG* Genetic Variants with Periodontitis and Type 2 Diabetes Mellitus in a Brazilian Population

**DOI:** 10.3390/ijms24076760

**Published:** 2023-04-05

**Authors:** Thamiris Cirelli, Ingra G. Nicchio, Diego G. Bussaneli, Bárbara R. Silva, Rafael Nepomuceno, Silvana R. P. Orrico, Joni A. Cirelli, Letícia H. Theodoro, Silvana P. Barros, Raquel M. Scarel-Caminaga

**Affiliations:** 1 Department of Dentistry, School of Dentistry, University Center—UNIFAE, São João da Boa Vista 13870-377, SP, Brazil; 2 Department of Diagnosis and Surgery, School of Dentistry at Araraquara, São Paulo State University—UNESP, Araraquara 14801-903, SP, Brazil; 3 Department of Morphology, Genetics, Orthodontics and Pediatric Dentistry, School of Dentistry at Araraquara, São Paulo State University—UNESP, Araraquara 14801-903, SP, Brazil; 4 Advanced Research Center in Medicine, Union of the Colleges of the Great Lakes—UNILAGO, São José do Rio Preto 15030-070, SP, Brazil; 5 Department of Diagnosis and Surgery, School of Dentistry at Araçatuba, São Paulo State University—UNESP, Araçatuba 16015-050, SP, Brazil; 6 Department of Periodontology, School of Dentistry, University of North Carolina at Chapel Hill—UNC, Chapel Hill, NC 27599, USA

**Keywords:** peroxisome proliferator-activated receptors, single nucleotide polymorphisms, periodontitis, type 2 diabetes mellitus, obesity, dyslipidemias

## Abstract

The *peroxisome proliferator-activated receptor gamma* (*PPARG*) gene encodes a transcription factor involved in the regulation of complex metabolic and inflammatory diseases. We investigated whether single nucleotide polymorphisms (SNPs) and haplotypes of the *PPARG* gene could contribute with susceptibility to develop periodontitis alone or together with type 2 diabetes mellitus (T2DM). Moreover, we evaluated the gene–phenotype association by assessing the subjects’ biochemical and periodontal parameters, and the expression of *PPARG* and other immune response–related genes. We examined 345 subjects with a healthy periodontium and without T2DM, 349 subjects with moderate or severe periodontitis but without T2DM, and 202 subjects with moderate or severe periodontitis and T2DM. *PPARG* SNPs rs12495364, rs1801282, rs1373640, and rs1151999 were investigated. Multiple logistic regressions adjusted for age, sex, and smoking status showed that individuals carrying rs1151999-GG had a 64% lower chance of developing periodontitis together with T2DM. The CCGT haplotype increased the risk of developing periodontitis together with T2DM. The rs1151999-GG and rs12495364-TC were associated with reduced risk of obesity, periodontitis, elevated triglycerides, and elevated glycated hemoglobin, but there was no association with gene expression. Polymorphisms of the *PPARG* gene were associated with developing periodontitis together with T2DM, and with obesity, lipid, glycemic, and periodontal characteristics.

## 1. Introduction

Periodontitis is a complex chronic immunoinflammatory disease of the oral cavity caused by dysbiosis of the oral microbiota [1]. It is characterized by a broad spectrum of systemic implications, including increased cardiovascular risk [2], and often coexists with several chronic metabolic diseases, such as T2DM, obesity, and metabolic syndrome [3]. T2DM is a complex endocrine and metabolic disease caused by defects in insulin secretion or insulin resistance due to genetic and/or environmental factors [3]. An important role in the evolution of many inflammatory diseases is attributed to reactive oxygen species (ROS), and changes in the activity of antioxidants in periodontal disease are influenced by systemic conditions. Furthermore, oxidative status markers and proinflammatory biomarkers are modified in the saliva of diabetic patients [4].

The peroxisome proliferator-activated receptors (PPARs) are ligand-activated transcription factors involved in a wide variety of regulatory functions. Dysregulation of PPAR-gamma (PPAR-γ) is linked to the development of complex diseases, such as obesity, type 2 diabetes mellitus (T2DM), atherosclerosis, osteoporosis [5], and Alzheimer’s disease [6]. PPAR-γ activation was reported to downregulate periodontal bone resorption [7], as mentioned by Schaefer et al., 2013 [8]; PPAR-γ was also implicated in the pathology of numerous co-morbidities of periodontitis, including obesity, diabetes and atherosclerosis [8]. Obese mice infected by *Porphyromonas gingivalis* showed the expression of PPAR-γ upregulated in the liver [9]; biopsies of gingiva from healthy individuals’ samples also had higher PPAR-γ expressions [10]. PPAR-γ agonist rosiglitazone controls blood glucose and has the ability to downregulate proinflammatory cytokine secretion through activation of PPAR-γ receptors, while also maintaining intracellular homeostasis by increasing insulin sensitivity, reducing oxidative stress, and suppressing inflammation [11]. T2DM, obesity, and periodontitis are strongly linked, and the mechanism that can explain this link is inflammation [12,13]. The inflammation underlying the relationship between periodontal disease and T2DM is mainly due to the individual’s response to chronic oral infection, which is influenced by her/his genetic makeup [14].

Single nucleotide polymorphisms (SNPs) represent the most common type of genetic variations among individuals. There is a lot of information about the genetic background of T2DM, including the high degree of polygenicity and the very small effect sizes of most genetic risk variants [15], but there is scarce evidence on the genetic association of some SNPs with susceptibility to develop periodontitis together with T2DM [14,16,17]. Given the identification of novel loci associated with common diseases such as T2DM, it is important to investigate their translation, despite several obstacles that complicate these kind of studies [15].

PPAR-γ plays a prominent role in regulating inflammatory reactions. In particular, it influences the differentiation of monocytes and attenuates the expression of pro-inflammatory mediators (e.g., tumor necrosis factor alpha [TNF-a], interleukin [IL]-1β, and IL-6 [18]). In this context, and because of the biological links between T2DM and periodontitis, as well as the many pieces of evidence pointing to rs1801282 SNP as a risk marker of T2DM [19,20], we hypothesize that this SNP and others in the *PPARG* gene might be markers of susceptibility to periodontitis together with T2DM. TagSNPs on the haplotype blocks of the *PPARG* gene evidenced by the data of validated SNPs from CEU + TSI were searched on the minor allele frequencies (MAF) higher than 0.05 in these CEU and TSI populations (CEU = individuals residing in Utah with Western and Northern European ancestry, and TSI = individuals from Tuscany in Italy). These data were obtained from the International HapMap Project database (www.hapmap.org). The selected tagSNPs were rs12495364, rs1373640, and rs1151999, in the respective first, third and fourth main haplotype blocks of the *PPARG* gene. For the selection of SNPs in the *PPARG* gene, we were also interested in those reported as potentially clinically relevant, as observed for the rs1151999 SNP that was associated with protection against Alzheimer’s disease [6]. In line with this prerogative, in the second haplotype block, we chose to investigate the rs1801282 SNP, which is a well-established risk marker of T2DM [19,20], and a relatively common SNP.

Despite the known association between periodontitis, T2DM and *PPARG*, and its biological importance in glucose metabolism and in the inflammatory response, nothing has been reported regarding the *PPARG* genetic variants (SNPs) in the risk and developing of periodontitis together with T2DM. We investigated whether SNPs and haplotypes of the *PPARG* gene could contribute, or not, with susceptibility to develop periodontitis, alone or together with T2DM. Moreover, we assessed gene–phenotype associations by using the biochemical, obesity, and periodontal data of subjects. We also evaluated whether circulating expression of *PPARG* and immune response–related genes are associated with the *PPARG* gene variants.

## 2. Results

We observed that the P + T2DM group presented higher visible plaque index than the Periodontitis group, which might contribute with a worse periodontal condition (higher percentage of sites with PPDi ≥ 5 mm and CALi between 4 and 5 mm and ≥6 mm). The P + T2DM group had the worst glycemic and lipid profiles and the physical examination parameters (Table 1).

Table 2 presents multiple logistic regressions adjusted for age, sex, and smoking status. The G allele of rs1151999 (G > T) in the *PPARG* gene was associated with a lower risk of developing periodontitis together with T2DM. Considering all subjects, those carrying rs1151999-GG had a 64% lower risk of developing periodontitis together with T2DM than individuals carrying the more common TT genotype (OR = 0.36, 95% CI = 0.18–0.73, *p* = 0.004). When separated by sex, male carriers of rs1151999-GG had an 89% lower risk of developing periodontitis together with T2DM (OR = 0.11, 95% CI = 0.03–0.39, *p* = 0.001). In the Heathy versus Periodontitis + P + T2DM group comparison, male carriers of rs1151999-GG were confirmed to be at significant lower risk (79%) of developing periodontitis with or without T2DM (OR = 021, 95% CI = 0.07–0.56, *p* = 0.002). We also found that ever-smokers carrying rs1151999-GG had a markedly lower risk of developing periodontitis together with T2DM (OR = 0.08, 95% CI = 0.02–0.37, *p* = 0.001) than those ever-smokers carrying rs1151999-TT. In the Periodontitis versus P + T2DM group comparison, besides rs1151999-GG, carriers of rs12495364-TC had about twice the risk to develop periodontitis together with T2DM, whether they are men (OR = 2.54; 95% CI = 1.25–5.19, *p* = 0.010) that is still significant after Bonferroni correction (*p* < 0.0125), or never-smokers (OR = 1.87; 95% CI = 1.08–3.21, *p* = 0.02).

We assessed the gene–phenotype associations with the periodontal, biochemical, and obesity parameters of the subjects. Based on linear regression, both rs1151999-GG and rs12495364-TC were significantly associated with reduced risk of hyperglycemia (elevated HbA1c), hyperlipidemia (elevated triglycerides), obesity (BMI and the waist-to-hip ratio) (Table 3), and periodontal parameters (bleeding on probing and the number of remaining teeth) (Table 4), even after adjustments for age, sex, and smoking status.

We evaluated whether the *PPARG* SNPs are transmitted together (as haplotypes), and it was confirmed by the strong LD among them (Figure S1B). The most frequent haplotype in the entire population was TCGG (about 33%), followed by TCAT (about 24.8%) (Figure S2). We performed multiple logistic regressions adjusted for age, sex, and smoking status similar to the analysis we performed for each SNP individually. For the Healthy versus P + T2DM group comparison, individuals carrying the CCGT haplotype were twice as likely to develop periodontitis together with T2DM (OR = 2.02; 95% CI = 1.28–3.16, *p* = 0.0027, Figure S2B). For the Healthy versus Periodontitis and P + T2DM group comparison, the CCGT haplotype was significantly associated with the disease phenotype (OR = 1.73; 95% CI = 1.21–2.48, *p* = 0.0029, Figure S2C).

**Figure 1:**
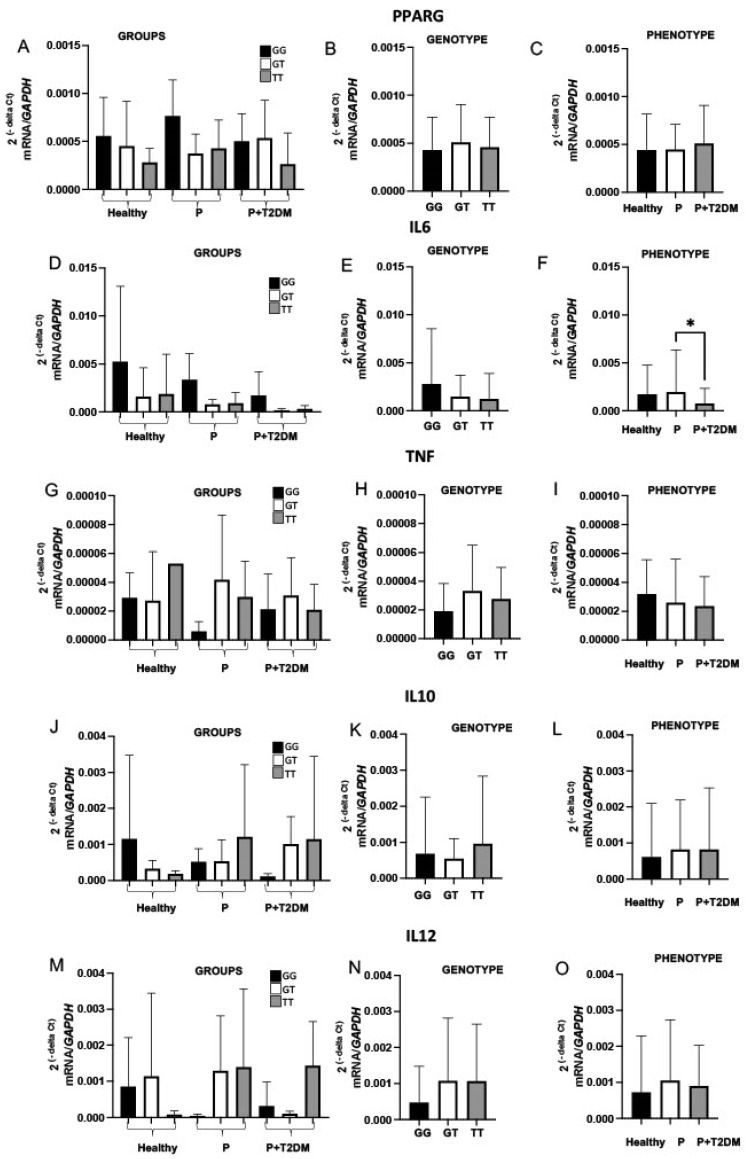
Expression of the *PPARG*, *IL6*, *TNF*, *IL10*, and *IL12* genes. The mean and standard error of 2^−ΔCt^ expression values of each gene after normalization by the *GAPDH*. In the first column (**A,D,G,J,M**), the subjects are divided according to the rs1151999 genotype, namely GG, GT, or TT, and the phenotype (Healthy, periodontitis [P], or periodontitis together with type 2 diabetes mellitus [P + T2DM], named Groups. In the second column (**B,E,H,K,N**), the subjects are divided based only on their genotypes, irrespective of the phenotypic classification. In the third column (**C,F,I,L,O**), the expression of each target gene is presented only according to the subjects’ phenotype, independently of the rs1151999 genotype. Comparisons among groups were made with the Kruskal-Wallis test, followed by Dunn’s test. * *p*-value ≤ 0.05.

Considering the more significant findings related to rs1151999 (Table 2), and that it is in LD with rs1801282 (D’ = 0.758, meaning approximately high LD, Figure S1B), we evaluated whether it could be associated with the circulating leukocyte expression of *PPARG*, *IL6*, *TNF*, *IL10* and *IL12* genes (Figure 1). Only individuals carrying rs1151999-GT had significant increased *IL6* gene expression (Figure 1F, *p*-value ≤ 0.05) compared with the other genotypes. No significant results were found for the other analyzed genes. Alternatively, we assessed the circulating leukocyte gene expression in each studied group, independently of the rs1151999 genotype, but again, no significant finding was observed.

## 3. Discussion

Although several studies investigating the role of SNPs in the *PPARG* gene in T2DM have been published, to our knowledge, this is the first study to investigate the potential contribution of the *PPARG* SNPs with the occurrence of periodontitis together with T2DM. The most investigated SNP in the *PPARG* gene regarding the association with T2DM is rs1801282 (Pro12Ala). Many case-control studies enrolling different ancestries have reported that the Pro12Ala (Ala12) variant is associated with protection against T2DM in East Asian (Japanese) [21,22], Greater Middle Eastern [23], and European ancestries such as Finnish [24], Czech [25] and White Scottish [26]. A meta-analysis from 73 studies, involving 62,250 cases and 69,613 controls, provided substantial evidence that the minor allele (G) of rs1801282 is associated with an approximately 18% decreased risk of developing T2DM under different genetic models and in different ethnicities [27]. In agreement, our study found the opposite allele (C) of rs1801282, composing the haplotype CCGT (the underlined C is the rs1801282-C), associated with two-fold odds to develop periodontitis together with T2DM (Figure S2B,C).

There have been few studies of how SNPs in the *PPARG* gene are related to periodontitis; most have investigated rs1801282. Researchers have shown a significant association between periodontitis and rs1801282 in obese elderly women, indicating a gene–gene or gene–environment interaction between periodontitis and rs1801282 [28]. A study enrolling a group of postmenopausal women with mild periodontitis showed no association between rs1801282 and periodontal clinical parameters [29]. In the present study, we did not find a significant association between rs1801282 and the development of periodontitis or periodontitis together with T2DM, but the rs1151999-GG was associated with a significantly reduced risk of developing periodontitis together with T2DM when considering all subjects or only men (Table 2). Besides the rs1151999-GG, other interesting results were found after stratifying the population by sex: the rs1373640 SNP was related to male carriers of the rs1373640-GA in the Periodontitis group, who showed a 47% lower risk of developing periodontitis (OR = 0.53, 95% CI = 0.30–0.92, *p* = 0.03), and male carriers of the rs12495364-TC had about twice the risk to develop periodontitis together with T2DM (Table 2). For a while, we were not able to directly explain these gene-phenotype results, but the literature demonstrates that PPAR-γ can influence androgen biosynthesis [30]. The PPAR-γ is a ligand-activated transcription factor expressed in adipose tissue, macrophages and ovaries that influences adipocyte differentiation, insulin sensitivity, and lipid metabolism [5,18]. There are differences in the distribution of adipose tissue between males and females, and the expression of PPAR-γ in perigonadal adipose tissues was significantly higher in females than in males. Further studies are necessary to investigate the functional role of these SNPs in the periodontal disease context, and to verify the possible influence of sex at the transcriptional or translational levels of the encoded proteins.

In the present study, the main significant findings are related to rs11519999-GG: considering the entire population, carriers of that minor allele in homozygosis were 64% less likely to develop periodontitis together with T2DM than carriers of rs11519999-TT. Consistently, carriers of rs1151999-G, together with three other *PPARG* SNPs, were strongly protected against Alzheimer’s disease when they did not carry the ε4 allele of *apolipoprotein E* (*APOE*) [6], indicating a gene–gene interaction. In the additive model, an increase in the minor allele of rs1151999-G marginally reduced the risk of high LDL cholesterol in women [31]. We also employed the additive model and found that rs1151999-GG reduced triglycerides, BMI, the waist-to-hip ratio, and HbA1c (Table 3). In agreement, PPAR-γ also regulates adipogenesis, and the rs1801282 SNP has been suggested to be a risk for obesity, since it was associated with BMI [32]. Moreover, for the first time the rs12495364-TC in the *PPARG* gene was associated with glycemic, lipid, obesity, and periodontal parameters (Table 3). On the other hand, further studies focusing on the investigation of genetic susceptibility to periodontitis might access whether, for example, the obesity phenotype could partially influence the results.

In addition to the *PPARG* gene–phenotype associations for T2DM and cardiovascular diseases, researchers have reported a *PPARG* SNP interaction with diet (fat intake), with a significant influence on bone mineral density (BMD) [33]. While rs1151999-G tends to reduce the risk of high LDL cholesterol [31], in the Framingham Offspring cohort the researchers found that the common allele rs1151999-T interacts with energy derived from dietary fat (*p* = 0.0004) as well as with the BMD of femoral neck in men [33]. There was a higher risk of vertebral fracture in Danish individuals heterozygous for the rs1151999 SNP (OR = 1.76; 95% CI = 1.18–2.63, *p* < 0.01) [5]. A recent meta-analysis enrolling 2,157,037 participants demonstrated that osteoporosis patients were more exposed to periodontitis (OR = 1.96; 95% CI, 1.50–2.54) [34]. Osteoporosis was significantly associated with severe alveolar crestal bone loss and the prevalence of periodontitis cases in postmenopausal Jordanian women [35]. In addition, osteoporotic women presented severe periodontitis with greater gingival inflammation, greater clinical attachment level and greater gingival recession than the women with normal BMD [36].

It is not uncommon to produce different results when analyzing SNPs as haplotypes rather than each one separately. Despite the importance of spanning the genetic region under study as much as possible to get better insight into the gene–disease association, only a few studies have focused on haplotypes of the *PPARG* gene. No previous report has evaluated the same combination of SNPs in the *PPARG* gene as we have done here. The only study focusing on periodontitis that evaluated haplotypes of the *PPARG* gene (A-C-C = rs2067819-A, rs1801282-C, and rs3856806-C) demonstrated a slight association with chronic periodontitis (OR = 1.53; 95% CI, 1.01–2.32, *p* = 0.04) [37]. The TCGG haplotype was most frequent in our population, followed by TCAT. Individuals carrying the CCGT haplotype were twice as susceptible to develop periodontitis together with T2DM, which we confirmed based on the occurrence of periodontitis when comparing the Periodontitis and P + T2DM groups with the Healthy group (OR = 1.73). Further discussion regarding this can be found in the Supplementary material [20,38,39,40,41,42,43,44,45,46,47,48,49,50].

Considering that rs1151999 is associated with the periodontitis together with T2DM phenotype, we examined its potential influence on the host’s inflammatory response. We noted only a tendency of higher *TNF*, *IL10* and *IL12* expression in PBMC of carriers of rs1151999-TT regardless of their phenotype, but no influence in the *PPARG* expression. Further discussion regarding this can be found in the Supplementary material. Again, our study is novel because no other researchers have evaluated the potential influence of this SNP on gene expression. Future studies enrolling more subjects who have been genotyped for this SNP, as well as SNPs in other exonic areas, could more accurately confirm these results.

The present study has a limitation of not investigating individuals solely affected by T2DM. We made this decision because of the high prevalence of periodontitis in individuals with T2DM, which motivated us to evaluate these diseases as comorbidities. Moreover, we were not able to investigate gene expression in a larger number of subjects, nor in gingival biopsies. However, we were interested in PBMC because information for these cells might reveal the potential influence of *PPARG* variants on the gene expression of circulating leukocytes. This is important because it is easier to assess venous blood at routine medical checkups compared with gingival biopsies. We acknowledge that additional studies are necessary to investigate other SNPs in the *PPARG* gene and the haplotypes, in larger and ethnically diverse cohorts of individuals affected by only T2DM or periodontitis, as well as by these two diseases as comorbidities, than we evaluated here (even though our experiment was sufficiently powered). It will also be important to verify the potential influence of sex and smoking in those populations, as well as parameters of obesity, such as BMI, and to evaluate the functionality of SNPs, mainly haplotypes, at the transcriptional and translational levels after inflammatory stimuli.

## 4. Materials and Methods

### 4.1. Subject Selection

**Figure 2:**
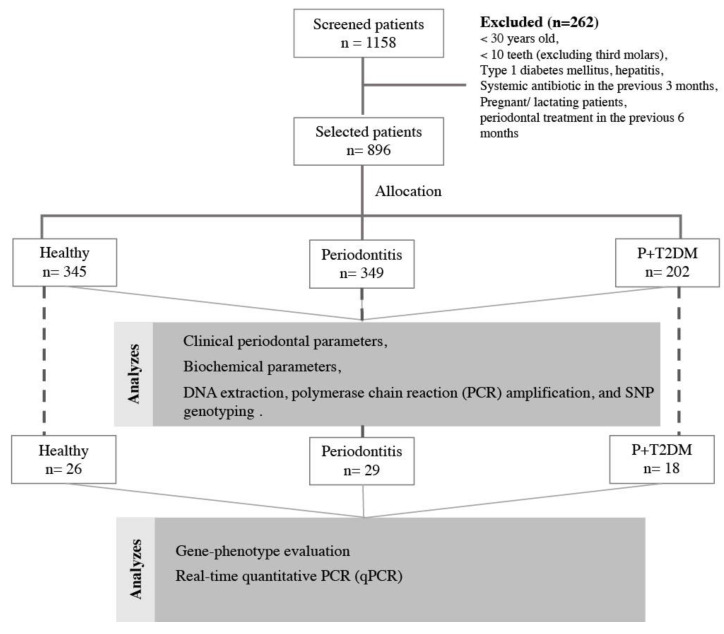
Flowchart of the study.

Figure 2 shows the flowchart of the study. From the pool of individuals at the School of Dentistry at Araraquara, São Paulo State University (UNESP), from the southeast region of Brazil, the total number of screened participants was 1158, of which 269 were excluded because of the following exclusion criteria: younger than 30 years; with less than 10 teeth (excluding third molars); with important systemic diseases, such as type 1 diabetes mellitus, hepatitis, HIV (human immunodeficiency virus) infection, and anemia; participants with reports of systemic antibiotics in the previous three months, immunosuppressive chemotherapy, chronic use of anti-inflammatory drugs, pregnant or lactating subjects; or who received periodontal treatment in the previous six months. A total of 896 unrelated participants were selected, and they were required to have some periodontal clinical and biochemical evaluations, as described below.

Each participant completed a questionnaire about their smoking status. We used the answers to classify them as never-smokers or ever-smokers (current smokers + former smokers). Regarding smoking status, participants were classified as a smoker when they reported smoking on a daily basis for at least one year with a mean of 20 cigarettes per day, and a former smoker when they had stopped smoking for at least one year [16,51]. Each participant also self-reported their skin color, as white (67.4%), black (10.83%), brown (19.31%) or mulatto (2.46%). We assessed the clinical periodontal parameters—interproximal probing pocket depth (PPDi), interproximal clinical attachment level (CALi), bleeding on probing (BOP), the visible plaque index, and marginal bleeding—by utilizing a University of North Carolina-15 periodontal probe at four sites (interproximal sites) around each tooth [16,17,51]. We followed the Centers for Disease Control and Prevention/American Academy of Periodontology (CDC/AAP) criteria to define periodontitis [52].

Peripheral blood was collected from all subjects in the morning after they had fasted for at least 8 h. After that, each subject was submitted to the periodontal examination. We used these samples to assess biochemical parameters. We assessed the glycemic profile, including fasting glycemia (using the modified Bondar and Mead method, Labtest Kit), glycated hemoglobin (HbA1c, using the turbidimetric inhibition immunoassay method, Roche), and insulin (using a chemiluminescence method); and the lipid profile, including triglycerides (using the enzymatic Trinder method, Labtest Kit), total cholesterol (using the enzymatic Trinder method, Labtest Kit), high-density lipoprotein cholesterol (HDL, using the enzymatic method, Labtest Kit), very-low-density lipoprotein cholesterol (VLDL), and low-density lipoprotein cholesterol (LDL). We used the Friedewald equation to determine the lipid parameters [53].

The T2DM diagnosis was obtained from their physicians and confirmed by the blood biochemical parameters tested on the day of the periodontal examination, according to the American Diabetes Association (ADA) parameters, fasting plasma glucose concentration ≥ 126 mg/dL (7.0 mmol/L) and/or an HbA1c ≥ 6.5% [54]. Based on the periodontal status and glycemic profile of each individual, we divided the subjects into three groups: 345 individuals with healthy periodontium or mild periodontitis and without T2DM (the Healthy group); 349 individuals with moderate or severe periodontitis and without T2DM (the Periodontitis group); and 202 individuals with moderate or severe periodontitis as well as T2DM (P + T2DM group). The inclusion and exclusion criteria have been described in previous studies [16,17,51].

This study was performed in accordance with the Declaration of Helsinki of 1964, as revised in 1983, and was approved by the Ethics in Human Research Committee of the Araraquara School of Dentistry (UNESP; CAAE 26839019.6.0000.5416), All participants gave oral and written consent to participate in the study. To ensure our experiment was sufficiently powered to evaluate the association of *PPARG* SNPs with periodontitis and T2DM, we calculated the required sample size by using the G*Power Calculator (version 3.182, Heinrich Heine University, Düsseldorf, Germany). We considered the following parameters: logistic regression; two tails; an odds ratio (OR) of 1.5; Pr (Y = 1/X = 1), H0 = 0.2; α of 0.0125 (0.05 divided by four SNPs); and 80% power, R2 = 0. We found that each group should contain at least 159 subjects.

### 4.2. DNA Extraction, Polymerase Chain Reaction (PCR) Amplification, and SNP Genotyping

Genomic DNA of each subject, obtained at least 1 h after they had brushed their teeth, was obtained from buccal epithelial cells after a mouthwash with glucose (3%) for 2 min. We extracted the DNA of each subject using standard protocols involving proteinase K digestion, 8 M ammonium acetate, and subsequent ethanol precipitation [55]. We quantified the genomic DNA with a NanoDrop^®^ 2000 spectrophotometer (Thermo Fisher Scientific, Waltham, MA, USA) and a Qubit^®^ 2.0 fluorometer (Thermo Fisher Scientific, Waltham, MA, USA). We used samples showing an A_260/280_ ratio between 1.7 and 2.0. We genotyped the rs12495364, rs1801282, rs1373640, and rs1151999 SNPs of the *PPARG* gene with 0.63 μL of the genotyping-specific TaqMan assay (Applied Biosystems, Thermo Fisher Scientific, Waltham, MA, USA) (including forward and reverse primers and VIC- and FAM-labeled probes, detailed in Table S1) as described previously [56].

### 4.3. Gene-Phenotype Evaluation

To obtain insight into the influence of the rs1151999 SNP on the expression of *PPARG* and pro- and anti-inflammatory genes, we recalled some individuals carrying each rs1151999 genotype (GG, GT or TT), and repeated the physical, biochemical and periodontal evaluations. Because we were interested in assessing the potential influence of rs1151999 on gene expression, we calculated the sample size with the DDS Research sample size calculator (SPH Analytics, Alpharetta, GA, USA), utilizing gene expression values from a previous study [57]. Based on this calculation, we required at least six subjects carrying each genotype (GG, GT, and TT) to have the ability to detect significant (at the 80% confidence level) differences of 0.3 units in the average gene expression values, with an estimated variation of 0.6 units. Fortunately, we were able to increase from the minimum sample size of 54 subjects to a total of 73 individuals, (Healthy, *n* = 26; P, *n* = 29; and P + T2DM, *n* = 18). At the same time blood was collected from each volunteer for the biochemical analyses, peripheral blood mononuclear cells (PBMC) were obtained by a Histopaque density gradient (Histopaque 1077 over Histopaque 1119, Sigma-Aldrich Co., St. Louis, MO, USA). After centrifugation (700× *g* for 30 min at room temperature), we washed PBMC twice with saline. Then, we resuspended PBMC in TRIzol and followed the manufacturer’s instructions to isolate total RNA. We determined the concentration and purity of total RNA with a UV microvolume spectrophotometer (Nanovue Plus, GE Health Sciences, Chicago, IL, USA).

We synthesized complementary DNA (cDNA) using 500 ng of total RNA and the High-Capacity Reverse Transcriptase Kit (Applied Biosystems, Foster City, CA, USA) according to the manufacturer’s instructions. We performed real-time quantitative PCR (qPCR) with TaqMan gene expression assays (Applied Biosystems, Thermo Fisher Scientific, Waltham, MA, USA) in duplicate to detect the target genes *PPARG* (Hs01115513_m1), *IL6* (Hs00174131_m1), *TNFA* (Hs01113624_g1), *IL10* (Hs00961622_m1), and *IL12* (Hs01073447_m1), and the endogenous control gene *GAPDH* (Hs02758991_g1). The thermocycling conditions in the StepOne Plus machine (Thermo Fisher Scientific) were: initial denaturation for 10 min at 95 °C, followed by 40 cycles of 15 s at 95 °C and 1 min at 60 °C.

### 4.4. Statistical Analyses

We have presented demographics, and periodontal, biochemical, and physical parameters, as the median and interquartile range (IQR) for numerical data, and as distribution frequencies for categorical data. We evaluated differences in the frequencies using the Kruskal-Wallis test, followed by Dunn’s test (age, periodontal, biochemical, and physical data), or the chi-square test (sex and smoking data), using SPSS Statistics (version 20, IBM Corp., Armonk, NY, USA) and GraphPad Prism (version 5.01, GraphPad Software, San Diego, CA, USA). To detect differences in the allele and genotype distributions of each SNP between the groups, and to test the Hardy-Weinberg equilibrium, we utilized the PLINK software (version 1.9, Harvard University, Cambridge, MA, USA) [58], as can be viewed in Table S2.

We determined the association between genotypes and disease phenotypes using multivariate logistic regression analyses assuming the additive model and adjusting for age, sex, and smoking status (as confounding factors), when we considered the entire population. We have expressed the effect size as an OR with a 95% confidence interval (CI). Furthermore, we stratified the same analysis (i) by sex, to identify the sex-specific effects of each SNPs on the pathological phenotype, but adjusting multiple logistic regressions by age and smoking; and (ii) by smoking status, adjusting the multiple logistic regressions by age and sex. To assess the gene–clinical–phenotype interrelationship, we performed multiple linear regressions to verify the independent association of each SNP with periodontal parameters (the number of teeth remaining, marginal bleeding, PPDi, and CALi) and with glycemic, lipid, and obesity parameters (fasting glucose, insulin level, HbA1c, triglycerides, total cholesterol, HDL, LDL, body mass index (BMI), and the waist-to-hip ratio). We used STATA (version 12.0, Stata Corporation, College Station, TX, USA) for logistic and linear regressions. We determined experiment-wise significance (*p* < 0.0125) by using Bonferroni’s correction because we genotyped four SNPs (0.05/4 = 0.0125).

We used the web-based tool SNPStats (www.SNPstats.com) [59] to calculate the linkage disequilibrium (LD) between the investigated SNPs to determine any co-segregation and to estimate the haplotype frequency. We used logistic regressions (adjusted for age, sex, and smoking status) to test associations between haplotypes and diseased phenotypes. We used the Expression Suite software (version 1.1, Applied Biosystems, Foster City, CA, USA) to normalize the cycle threshold (Ct) value of each target gene to *GAPDH* gene expression, by the 2^−ΔCt^ method. We used the Kruskal-Wallis test followed by the Dunn’s test for statistical comparison (using GraphPad Prism 5.01). We considered *p* < 0.05 to be significant.

## 5. Conclusions

In conclusion, our multiple logistic regressions adjusted for age, sex, and smoking status indicate that rs1151999-GG may contribute to reducing the chance to develop periodontitis together with T2DM, while the CCGT haplotype increases this disease phenotype. The rs1151999-GG and rs12495364-TC together are associated with reduced risk of obesity, periodontitis, high triglycerides, and high HbA1c, but there is no association with gene expression in circulating leukocytes.

## Figures and Tables

**Table 1:** Comparison of demographic characteristics, and periodontal, biochemical, and physical parameters among the groups.

Demographic Characteristics	Healthy	Periodontitis	Periodontitis + T2DM
	*n* = 345	*n* = 349	*n* = 202
Age—Median (IQR)	41.0 (35.0–50.0) ^a^	49.0 (43.0–55.0) ^b^	56.0 (50.0–62.0) ^c^
Sex *n* (%) *			
Males	114 (32.5)	143 (40.9)	85 (42.1)
Females	233 (67.5)	206 (59.1)	117 (57.9)
Smoking habits (%) *			
Never	268 (77.7)	224 (64.2)	114 (56.4)
Ever	77 (22.3)	125 (35.8)	88 (43.6)
Periodontal Parameters—Median (IQR)			
Number of teeth	27.0 (24.0–28.0) ^a^	24.0 (20.0–26.0) ^b^	21.0 (16.0–25.0) ^c^
Visible plaque (% of sites)	20.4 (8.2–36.6) ^a^	43.4 (22.0–67.9) ^b^	56.7 (36.5–77.5) ^c^
Marginal bleeding (% of sites)	4.2 (0.0–11.8) ^a^	11.0 (3.1–28.3) ^b^	36.5 (21.8–52.8) ^c^
BOP (% of sites)	1.0 (0.0–4.7) ^a^	37.0 (20.6–55.6) ^b^	52.3 (28.1–72.4) ^c^
PPDi ≤ 4 mm (% of sites)	100.0 (100.0–100.0) ^a^	91.3 (80.9–97.0) ^b^	87.5 (68.4–97.8) ^b^
PPDi ≥ 5 mm (% of sites)	0.0 (0.0–0.0) ^a^	8.6 (2.9–19.1) ^b^	12.5 (2.2–31.6) ^b^
CALi ≤ 3 mm (% of sites)	110.0 (99.1–100.0) ^a^	72.4 (55.5–85.4) ^b^	58.3 (33.3–80.4) ^c^
CALi = 4–5 mm (% of sites)	0.0 (0.0–0.93) ^a^	17.9 (10.1–28.3) ^b^	24.1 (11.2–34.8) ^b^
CALi ≥ 6 mm (% of sites)	0.0 (0.0–2.1) ^a^	6.3 (2.1–15.4) ^b^	11.1 (2.5–28.8) ^b^
Biochemical and Physical data—Median (IQR)			
Fasting blood glucose (mg/dL)	92.0 (86.0–96.0) ^a^	94.5 (89.0–99.0) ^a^	153.1 (118.0–217.0) ^b^
HbA1c (percentage)	5.4 (5.2–5.6) ^a^	5.6 (5.3–5.6) ^a^	7.9 (6.6–9.7) ^b^
Insulin (UI/mL)	7.4 (3.6–37.8) ^a^	9.9 (4.1–32.4) ^a^	13.8 (3.2–161.0) ^b^
Total cholesterol (mg/dL)	174.0 (152.0–194.0) ^a^	189.0 (160.5–208.5) ^a,b^	191.0 (160.0–227.0) ^b^
HDL cholesterol (mg/dL)	54.0 (40.0–61.0) ^a^	51.5 (42.0–60.2) ^a^	44.0 (37.0–55.5) ^b^
LDL cholesterol (mg/dL)	97.0 (78.0–121.2) ^a^	98.4 (79.4–116.0) ^a^	108.2 (70.9–142.5) ^a^
Triglycerides (mg/dL)	105.0 (81.0–156.0) ^a^	138.5 (94.5–203.0) ^b^	155.0 (105.8–240.3) ^b^
BMI (kg/m^2^)	27.1 (22.4–29.7) ^a^	26.2 (24.2–29.5) ^a^	29.5 (26.3–33.4) ^b^
Waist/hip ratio (cm)	0.87 (0.78–0.95) ^a^	0.89 (0.83–0.97) ^a^	0.95 (0.91–1.0) ^b^

Abbreviations: BOP, bleeding on probing; CALi, interproximal clinical attachment level; IQR, interquartile range (25th percentile to 75th percentile); P, periodontitis; PPDi, interproximal probing pocket depth. ^a^, ^b^, ^c^ = different letters indicate significant differences among groups (*p* < 0.05). * Significant difference in sex and smoking status distribution between groups (*p* < 0.0001). Categorical sex and smoking status intergroup comparisons were made using the chi-square test. Age, and periodontal, biochemical, and physical parameters, were analyzed by the Kruskal–Wallis test, followed by Dunn’s test.

**Table 2:** Multiple logistic regressions adjusted for age, sex, and smoking status and stratified by sex and smoking status.

*PPARG* SNP	Healthy vs. Periodontitis		Healthy vs. P + T2DM		Periodontitis vs. P + T2DM		Healthy vs. Periodontitis + P + T2DM	
rs12495364 (T > C)								
All subjects ^†^	Adjusted OR ^†^		Adjusted OR ^†^		Adjusted OR ^†^		Adjusted OR ^†^	
TT	Ref		Ref					
TC	0.75 (0.56–1.05)	0.10	1.53 (0.94–2.47)	0.08	1.50 (0.96–2.33)	0.07	1.57 (0.04–2.36)	0.05
CC	0.62 (0.36–1.08)	0.09	1.73 (0.79–3.80)	0.17	1.51 (0.76–3.02)	0.24	1.67 (0.87–3.19)	0.12
Male ^‡^	Adjusted OR ^‡^		Adjusted OR ^‡^		Adjusted OR ^‡^		Adjusted OR ^‡^	
CC	Ref		Ref		Ref		Ref	
TC	0.62 (0.35–1.12)	0.11	2.13 (0.89–5.05)	0.09	**2.54 (1.25–5.19)**	0.010 *	**2.29 (1.18–4.44)**	**0.014**
TT	0.84 (0.33–2.14)	0.72	1.24 (0.32–4.84)	0.75	1.86 (0.62–5.58)	0.27	1.73 (0.61–4.90)	0.29
Female ^‡^								
CC	Ref		Ref		Ref		Ref	
TC	1.02 (0.67–1.55)	0.90	1.32 (0.73–2.39)	0.35	1.07 (0.61–1.88)	0.81	1.24 (0.74–2.09)	0.40
TT	1.42 (0.65–3.12)	0.38	2.14 (0.79–5.75)	0.13	1.35 (0.56–3.31)	0.51	1.67 (0.73–3.84)	0.22
Never Smoking ^§^	Adjusted OR ^§^		Adjusted OR ^§^		Adjusted OR ^§^		Adjusted OR^§^	
CC	Ref		Ref		Ref		Ref	
TC	0.78 (0.53–1.17)	0.24	1.70 (0.96–3.01)	0.07	**1.87 (1.08–3.21)**	**0.02**	**1.87 (1.13–3.09)**	**0.02**
TT	1.11 (0.54–2.27)	0.77	2.04 (0.82–5.07)	0.13	1.94 (0.83–4.47)	0.12	2.17 (0.99–3.09)	0.20
Ever Smoking ^§^								
CC	Ref		Ref		Ref		Ref	
TC	1.01 (0.57–2.12)	0.76	1.32 (0.52–3.41)	0.56	1.12 (0.51–2.48)	0.77	1.23 (0.59–2.55)	0.57
TT	1.25 (0.42–3.76)	0.69	1.35 (0.25–7.16)	0.73	1.05 (0.28–3.86)	0.94	1.03 (0.30–3.59)	0.95
rs1801282 (C > G)								
All subjects ^†^	Adjusted OR ^†^		Adjusted OR ^†^		Adjusted OR ^†^		Adjusted OR ^†^	
CC	Ref		Ref		Ref		Ref	
CG	0.75 (0.48–1.15)	0.19	0.77 (0.43–1.39)	0.39	0.97 (0.56–1.68)	0.92	0.87 (0.53–1.45)	0.60
GG	0.27 (0.04–1.62)	0.15	1.01 (0.21–4.84)	0.99	3.46 (0.61–19.74)	0.16	1.68 (0.44–6.51)	0.45
Male ^‡^	Adjusted OR ^‡^		Adjusted OR ^‡^		Adjusted OR ^‡^		Adjusted OR ^‡^	
CC	Ref		Ref		Ref		Ref	
CG	0.52 (0.26–1.02)	0.06	0.64 (0.25–1.61)	0.34	1.51 (0.63–3.59)	0.35	1.08 (0.49–2.36)	0.84
GG	empty		0.48 (0.04–6.47)	0.58	empty		2.36 (0.19–29.28)	0.50
Female ^‡^								
CC	Ref		Ref		Ref		Ref	
CG	0.99 (0.57–1.74)	0.99	0.92 (0.43–1.99)	0.84	0.72 (0.34–1.50)	0.38	0.79 (0.39–1.53)	0.47
GG	0.53 (0.08–3.28)	0.49	1.28 (0.19–8.34)	0.79	1.92 (0.26–14.22)	0.52	1.36 (0.25–7.37)	0.71
Never Smoking ^§^	Adjusted OR ^§^		Adjusted OR ^§^		Adjusted OR ^§^		Adjusted OR ^§^	
CC	Ref		Ref		Ref		Ref	
CG	0.70 (0.42–1.17)	0.18	0.83 (0.42–1.67)	0.61	1.07 (0.55–2.08)	0.84	1.01 (0.55–2.83)	0.98
GG	0.25 (0.02–2.42)	0.23	1.66 (0.30–9.06)	0.56	5.89 (0.59–58.4)	0.13	2.69 (0.57–12.64)	0.21
Ever Smoking ^§^								
CC	Ref		Ref		Ref		Ref	
CG	0.87 (0.39–1.93)	0.74	0.71 (0.23–2.15)	0.54	0.95 (0.34–2.71)	0.94	0.72 (0.28–1.84)	0.49
GG	0.35 (0.02–6.43)	0.51	0.16 (0.007–3.04)	0.22	1.17 (0.06–22.51)	0.91	0.43 (0.03–6.04)	0.53
rs1373640 (A > G)								
All subjects ^†^	Adjusted OR ^†^		Adjusted OR ^†^		Adjusted OR ^†^		Adjusted OR ^†^	
GG	Ref		Ref					
GA	**0.72 (0.87–0.99)**	**0.05**	0.74 (0.47–1.16)	0.19	1.01 (0.67–1.53)	0.95	0.85 (0.58–1.25)	0.42
AA	1.11 (0.55–2.26)	0.76	0.87 (0.33–2.29)	0.77	0.98 (0.41–2.33)	0.96	0.95 (0.42–2.11)	0.89
Male ^‡^	Adjusted OR ^‡^		Adjusted OR ^‡^		Adjusted OR ^‡^		Adjusted OR ^‡^	
GG	Ref		Ref		Ref		Ref	
GA	**0.53 (0.30–0.92)**	**0.03**	0.87 (0.38–1.97)	0.75	1.30 (0.66–2.56)	0.44	1.16 (0.62–2.19)	0.47
AA	1.72 (0.56–5.27)	0.34	3.65 (0.81–16.41)	0.09	2.25 (0.70–7.24)	0.17	2.77 (0.92–8.39)	0.71
Female ^‡^								
GG	Ref		Ref		Ref		Ref	
GA	0.85 (0.57–1.28)	0.46	0.70 (0.40–1.23)	0.22	0.84 (0.49–1.43)	0.53	0.72 (0.44–1.15)	0.17
AA	0.82 (0.32–2.11)	0.68	0.24 (0.04–1.25)	0.09	0.35 (0.0–1.73)	0.20	0.28 (0.06–1.28)	0.20
Never Smoking ^§^	Adjusted OR ^§^		Adjusted OR ^§^		Adjusted OR ^§^		Adjusted OR^§^	
GG	Ref		Ref		Ref		Ref	
GA	0.76 (0.51–1.12)	0.16	0.63 (0.37– 1.09)	0.10	0.75 (0.44–1.26)	0.28	0.65 (0.40–1.06)	0.09
AA	0.94 (0.41–2.11)	0.86	0.40 (0.11–1.44)	0.16	0.68 (0.21–2.23)	0.53	0.59 (0.21–1.77)	0.35
Ever Smoking ^§^								
GG	Ref		Ref		Ref		Ref	
GA	0.62 (0.34–1.16)	0.14	0.99 (0.43–2.27)	0.98	1.67 (0.81–3.42)	0.16	1.31 (0.68–2.53)	0.41
AA	1.99 (0.39–10.01)	0.40	4.62 (0.72–29.66)	0.11	2.19 (0.57–8.43)	0.25	2.24 (0.64–7.87)	0.21
rs1151999 (G > T)								
All subjects ^†^	Adjusted OR ^†^		Adjusted OR ^†^		Adjusted OR ^†^		Adjusted OR ^†^	
TT	Ref		Ref					
TG	**0.69 (0.49–0.99)**	**0.05**	0.72 (0.44–1.15)	0.17	0.93 (0.61–1.42)	0.76	0.77 (0.52–1.14)	0.20
GG	0.68 (0.43–1.1.07)	0.10	**0.36 (0.18–0.73)**	0.004 *	0.67 (0.36–1.24)	0.21	0.49 (0.27–1.19)	0.20
Male ^‡^	Adjusted OR ^‡^		Adjusted OR ^‡^		Adjusted OR ^‡^		Adjusted OR ^‡^	
TT	Ref		Ref		Ref		Ref	
TG	0.56 (0.29–1.04)	0.07	**0.35 (0.14–0.87)**	**0.02**	0.51 (0.25–1.03)	0.06	**0.42 (0.21–0.82)**	**0.02**
GG	0.74 (0.35–1.60)	0.35	**0.11 (0.03–0.39)**	0.001 *	**0.28 (0.11–0.77)**	**0.014**	**0.21 (0.07–0.56)**	0.002 *
Female ^‡^								
TT	Ref		Ref		Ref		Ref	
TG	0.77 (0.50–1.19)	0.25	0.99 (0.56–1.76)	0.98	1.29 (0.79–2.23)	0.35	1.08 (0.65–1.78)	0.75
GG	0.59 (0.33–1.07)	0.08	0.63 (0.28–1.43)	0.27	1.21 (0.54–2.73)	0.64	0.83 (0.40–1.73)	0.63
Never Smoking ^§^	Adjusted OR ^§^		Adjusted OR ^§^		Adjusted OR ^§^		Adjusted OR ^§^	
TT	Ref		Ref		Ref		Ref	
TG	0.77 (0.51–1.15)	0.20	0.94 (0.53–1.65)	0.82	0.97 (0.56–1.64)	0.89	0.85 (0.52–1.40)	0.54
GG	0.61 (0.35–1.07)	0.09	0.59 (0.27–1.30)	0.19	1.27 (0.58–2.78)	0.59	0.87 (0.43–1.75)	0.71
Ever Smoking ^§^								
TT	Ref		Ref		Ref		Ref	
TG	0.54 (0.27–1.10)	0.09	**0.32 (0.12–0.82)**	**0.02**	0.77 (0.36–1.64)	0.49	0.59 (0.29–1.18)	0.14
GG	0.79 (0.34–1.82)	0.59	**0.08 (0.02–0.37)**	0.001 *	**0.20 (0.06–0.62)**	0.005 *	**0.16 (0.05–0.47)**	0.001 *

Abbreviations: P + T2DM = periodontitis together with type 2 diabetes mellitus; Adj OR (95% CI) = Adjusted odds ratio (95% confidence interval); Ref = reference or more frequent single nucleotide polymorphism. Never smokers = subjects who never smoked; ever smokers = current smokers + former smokers. * Indicates statistical significance after Bonferroni correction (*p* < 0.0125). ^†^ ORs with 95% CIs were estimated by multiple logistic regression models after controlling for age, sex, and smoking status. ^‡^ ORs with 95% CIs were estimated by multiple logistic regression models after controlling for age and smoking status. ^§^ ORs with 95% CIs were estimated by multiple logistic regression models after controlling for age and sex. **Bold font** indicates *p* < 0.05.

**Table 3:** Multiple linear regression analysis of all subjects for effects of single nucleotide polymorphisms on glycemic, lipid, and obesity parameters.

*PPARG* SNP	Fasting Glucose		Insulin		HbA1c		Triglycerides		Total Cholesterol		HDL		LDL		BMI (kg/m^2^)		Waist-To-Hip Ratio (cm)	
rs12495364	Adjusted β ^†^ (95% CI)	*p*-Value	Adjusted β ^†^ (95% CI)	*p*-Value	Adjusted β ^†^ (95% CI)	*p*-Value	Adjusted β ^†^ (95% CI)	*p*-Value	Adjusted β ^†^ (95% CI)	*p*-Value	Adjusted β ^†^ (95% CI)	*p*-Value	Adjusted β ^†^ (95% CI)	*p*-Value	Adjusted β ^†^ (95% CI)	*p*-Value	Adjusted β ^†^ (95% CI)	*p*-Value
TT	Ref		Ref		Ref		Ref		Ref		Ref		Ref		Ref		Ref	
TC	**−0.14** **(−0.26–−0.03)**	**0.008**	**−0.14** **(−0.28–−0.01)**	**0.03**	**−0.14** **(−0.25–−0.03)**	**0.01**	**−0.13** **(−0.25–−0.02)**	**0.01**	**−0.12** **(−0.23–−0.01)**	**0.03**	**−0.11** **(−0.23–−0.001)**	**0.04**	**−0.12** **(−0.24–−0.001)**	**0.04**	**−0.14** **(−0.25–−0.03)**	**0.01**	**−0.25** **(−0.38–−0.11)**	0.000 *
CC	−0.01 (−0.25–0.21)	0.88	0.19 (−0.26–0.29)	0.89	−0.06 (−0.29–0.16)	0.58	−0.04 (−0.28–0.20)	0.74	0.02 (−0.22–0.27)	0.84	0.006 (−0.24–0.26)	0.95	0.03 (−0.22–0.29)	0.80	−0.02 (−0.27–0.21)	0.82	−0.01 (−0.28–0.25)	0.88
rs1801282																		
CC	Ref		Ref		Ref		Ref		Ref		Ref		Ref		Ref		Ref	
CG	0.02 (−0.07–0.13)	0.58	−0.002 (−0.13–0.13)	0.97	0.004 (−0.09–0.10)	0.92	0.01 (−0.08–0.12)	0.71	0.02 (−0.08–0.12)	0.69	0.01 (−0.09–0.12)	0.75	0.008 (−0.10–0.12)	0.88	0.02 (−0.07–0.12)	0.68	−0.01 (−0.14–0.11)	0.80
GG	**0.21** **(0.01–0.41)**	**0.03**	0.14 (−0.09–0.39)	0.23	0.17 (−0.01–0.37)	0.07	0.13 (−0.07–0.34)	0.20	0.17 (−0.03–0.38)	0.10	0.17 (−0.03–0.39)	0.10	0.17 (−0.05–0.40)	0.13	0.20 (−0.01–0.43)	0.07	0.24 (−0.04–0.53)	0.09
rs1373640																		
GG	Ref		Ref		Ref		Ref		Ref		Ref		Ref		Ref		Ref	
GA	0.01 (−0.09–0.12)	0.74	−0.04 (−0.17–0.09)	0.56	−0.001 (−0.10–0.10)	0.98	−0.03 (−0.13–0.07)	0.57	−0.03 (−0.14–0.07)	0.55	−0.02 (−0.13–0.08)	0.66	−0.01 (−0.12–0.10)	0.83	−0.03 (−0.14–0.06)	0.48	−0.04 (−0.18–0.09)	0.52
AA	0.01 (−0.33–0.36)	0.92	−0.03 (−0.53–0.46)	0.89	0.04 (−0.29–0.37)	0.81	−0.01 (−0.37–0.34)	0.93	0.02 (−0.34–0.38)	0.90	0.01 (−0.36–0.38)	0.95	−0.01 (−0.43–0.40)	0.94	0.005 (−0.33–0.35)	0.97	−0.10 (−0.59–0.38)	0.68
rs1151999																		
TT	Ref		Ref		Ref		Ref		Ref		Ref		Ref		Ref		Ref	
TG	−0.005 (−0.11–0.10)	0.92	−0.07 (−0.22–0.07)	0.30	−0.04 (−0.14–0.06)	0.38	−0.07 (−0.19–0.06)	0.31	−0.04 (−0.16–0.09)	0.63	−0.03 (−0.16–0.10)	0.65	−0.04 (−0.18–0.09)	0.53	−0.09 (−0.21–0.04)	0.17	−0.14 (−0.29–0.01)	0.07
GG	−0.07 (−0.22–0.08)	0.34	−0.11 (−0.31–0.08)	0.28	**−0.15** **(−0.28–−0.003)**	**0.04**	**−0.19** **(−0.37–−0.02)**	**0.03**	−0.16 (−0.34–0.02)	0.08	−0.17 (−0.35–0.02)	0.07	−0.16 (−0.34–0.03)	0.10	**−0.21** **(−0.39–0.04)**	**0.02**	**−0.23** **(−0.44–0.02)**	**0.03**

Abbreviations: β, regression coefficient; BMI, body mass index; CI, confidence interval; HbA1C, glycated hemoglobin; HDL, high-density lipoprotein; LDL, low-density lipoprotein. * Indicates statistical significance after Bonferroni correction (*p* < 0.0125). ^†^ β the regression coefficient with 95% CI was estimated by multiple linear regression models after controlling for age, sex and smoking. **Bold font** indicates *p* < 0.05.

**Table 4:** Multiple linear regression analysis of all subjects for effects of single nucleotide polymorphisms on periodontal parameters.

*PPARG* SNP	Number of Remaining Teeth		Bleeding on Marginal Probing (% of Sites)		Bleeding on Pocket Probing (% of Sites)		PPDi ≥ 5 mm (% of Sites)		CALi ≥ 6 mm (% of Sites)	
rs12495364	Adjusted β * (95% CI)	p-value	Adjusted β * (95% CI)	*p*-Value	Adjusted β * (95% CI)	*p*-Value	Adjusted β * (95% CI)	*p*-Value	Adjusted β * (95% CI)	*p*-Value
TT	Ref		Ref		Ref		Ref		Ref	
TC	**−0.14** **(−0.26–−0.03)**	**0.008**	**−0.14** **(−0.28–−0.01)**	**0.03**	**−0.14** **(−0.25–−0.03)**	**0.01**	**−0.13** **(−0.25–−0.02)**	**0.01**	**−0.12** **(−0.23–−0.01)**	**0.03**
CC	−0.01 (−0.25–0.21)	0.88	0.19 (−0.26–0.29)	0.89	−0.06 (−0.29–0.16)	0.58	−0.04 (−0.28–0.20)	0.74	0.02 (−0.22–0.27)	0.84
rs1801282										
CC	Ref		Ref		Ref		Ref		Ref	
CG	0.02 (−0.07–0.13)	0.58	−0.002 (−0.13–0.13)	0.97	0.004 (−0.09–0.10)	0.92	0.01 (−0.08–0.12)	0.71	0.02 (−0.08–0.12)	0.69
GG	**0.21** **(0.01–0.41)**	**0.03**	0.14 (−0.09–0.39)	0.23	0.17 (−0.01–0.37)	0.07	0.13 (−0.07–0.34)	0.20	0.17 (−0.03–0.38)	0.10
rs1373640										
GG	Ref		Ref		Ref		Ref		Ref	
GA	0.01 (−0.09–0.12)	0.74	−0.04 (−0.17–0.09)	0.56	−0.001 (−0.10–0.10)	0.98	−0.03 (−0.13–0.07)	0.57	−0.03 (−0.14–0.07)	0.55
AA	0.01 (−0.33–0.36)	0.92	−0.03 (−0.53–0.46)	0.89	0.04 (−0.29–0.37)	0.81	−0.01 (−0.37–0.34)	0.93	0.02 (−0.34–0.38)	0.90
rs1151999										
TT	Ref		Ref		Ref		Ref		Ref	
TG	−0.03 (−0.09–0.02)	0.25	−0.006 (−0.07–0.05)	0.83	−0.01 (−0.06–0.04)	0.63	−0.02 (−0.07–0.04)	0.54	−0.01 (−0.07–0.04)	0.59
GG	**−0.07** **(−0.15–−0.002)**	**0.04**	**−0.08** **(−0.16–−0.12)**	**0.02**	−0.06 (−0.13–−0.01)	0.09	−0.07 (−0.14–−0.008)	0.08	−0.06 (−0.14–0.009)	0.09

Abbreviations: β, regression coefficient; CALi, interproximal clinical attachment level; CI, confidence interval; PPDi, interproximal probing pocket depth. * β the regression coefficient with 95% CI was estimated by multiple linear regression models after controlling for age, sex, and smoking status. **Bold font** indicates *p* < 0.05.

## Supplementary Materials

The following supporting information can be downloaded at: https://www.mdpi.com/article/10.3390/ijms24076760/s1. Additional discussion regarding the haplotypes of the *PPARG* gene and the expression of *PPARG* and other immune response-related genes in PBMC of subjects can be found in the Supplementary material, with the References [20,38,39,40,41,42,43,44,45,46,47,48,49,50].
