# Supplementary material for: Evidence Linking PPARG Genetic Variants with Periodontitis and Type 2 Diabetes Mellitus in a Brazilian Population
Source: Int J Mol Sci. 2023 Apr 5;24(7):6760. doi: 10.3390/ijms24076760 (PMC10095581; doi:10.3390/ijms24076760)
Supplement: Supplementary file 1 [file ijms-24-06760-s001.zip › 1_Supplementary material Discussion_SpecialIssue.pdf]

Supplementary material

Evidence linking PPARG genetic variants with periodontitis and type 2 diabetes mellitus in a Brazilian population

Thamiris Cirelli [1,2,*], Ingra G Nicchio [2,3], Diego G Bussaneli [3], Bárbara R Silva [2,3], Rafael Nepomuceno [2], Silvana R P Orrico [2,4], Joni A Cirelli [2], Letícia H Theodoro [5], Silvana P Barros [6], Raquel M Scarel-Caminaga [3]

Supplementary Discussion

The result that individuals carrying the CCGT haplotype were twice as susceptible to develop periodontitis together with T2DM makes sense: the rs12495364-C allele at the first position of the CCGT haplotype (Supplementary Figure 2C) is compatible with the increased risk of developing periodontitis together with T2DM in male carriers of rs12495364-TC (Table 2). Concerning the second position of the haplotype, the rs1801282-C allele confers high risk of T2DM [20]. Moreover, rs1151999-T at the fourth position is associated with a higher risk of developing periodontitis together with T2DM (Supplementary Figure 2). Indeed, rs1151999-T has an opposite effect to rs1151999-G, which reduced the risk of developing periodontitis together with T2DM (Table 2).

We examined the potential influence of the rs1151999 genotypes in the host's inflammatory response by evaluating the gene expression in the PBMC of a subpopulation of our study. To discuss the gene expression in the context of periodontitis, it would be better to investigate gene expression in gingival biopsies. Unfortunately, it is difficult to obtain gingival tissue from subjects because the criteria for collecting these biopsies after dental treatment must be met. Only one study has reported higher expression of the PPAR-γ protein in the gingival tissue of individuals with periodontitis, as well as in individuals with peri-implantitis, compared with individuals with a healthy periodontium [38]. Experimental periodontitis in rats has shown that PPAR-γ plays an anti-inflammatory role in experimental periodontitis, attenuating the production of pro-inflammatory cytokines and, consequently, the destruction of periodontal tissues [39]. Another study concerning experimental periodontitis in rats showed that PPAR-γ could inhibit inflammation, preventing bone resorption within 1-4 weeks, while long-term PPAR-γ activation (8 weeks) increased bone resorption, and PPAR-γ repression by an antagonist enhanced alveolar bone formation [38, 40, 41].

We investigated the expression of *PPARG* and other immune response–related genes in PBMC of subjects because several studies have used these cells to investigate the role of *PPARG* in the modulation of immunomodulatory mechanisms [42, 45-48, 51, 52]. In PBMC of overweight rats, there were differences in *PPARG* mRNA expression even in the early stages, showing similar patterns with the expression observed in liver cells and adipose tissue [49]. Such a study demonstrates that PBMC can be used as early predictors of obesity-related

homeostatic imbalance [49]. PBMC are also an experimental model of choice, obtained from individuals with asthma or atopic allergy after stimulation with lipopolysaccharide from *Escherichia coli*, to determine differences in the levels of transcripts and inflammatory proteins (including PPAR-γ) compared with healthy individuals [50].
