# Supplementary material for: Evidence Linking PPARG Genetic Variants with Periodontitis and Type 2 Diabetes Mellitus in a Brazilian Population
Source: Int J Mol Sci. 2023 Apr 5;24(7):6760. doi: 10.3390/ijms24076760 (PMC10095581; doi:10.3390/ijms24076760)
Supplement: Supplementary file 1 [file ijms-24-06760-s001.zip › 2_SupplementaryFigure.pdf]

Supplementary material

Evidence linking PPARG genetic variants with periodontitis and type 2 diabetes mellitus in a Brazilian population

Figure S1. Scheme of the haplotypes formed by the SNPs in the *PPARG* gene. (A) The gametic phase and base pair position on the Chromosome 3 of the *PPARG* SNPs considering the Genome Reference Consortium Human Build 38 (GRCh38.p12). (B) Results of linkage disequilibrium of the SNPs analyzed in pairs. Abbreviations: D = disequilibrium coefficient. D′ = the linkage disequilibrium coefficient D dividing it by the theoretical maximum value it can take (Dmax). r = Pearson′s correlation coefficient between allele frequencies. X^2 = Chi-squared test. n=number of subjects.

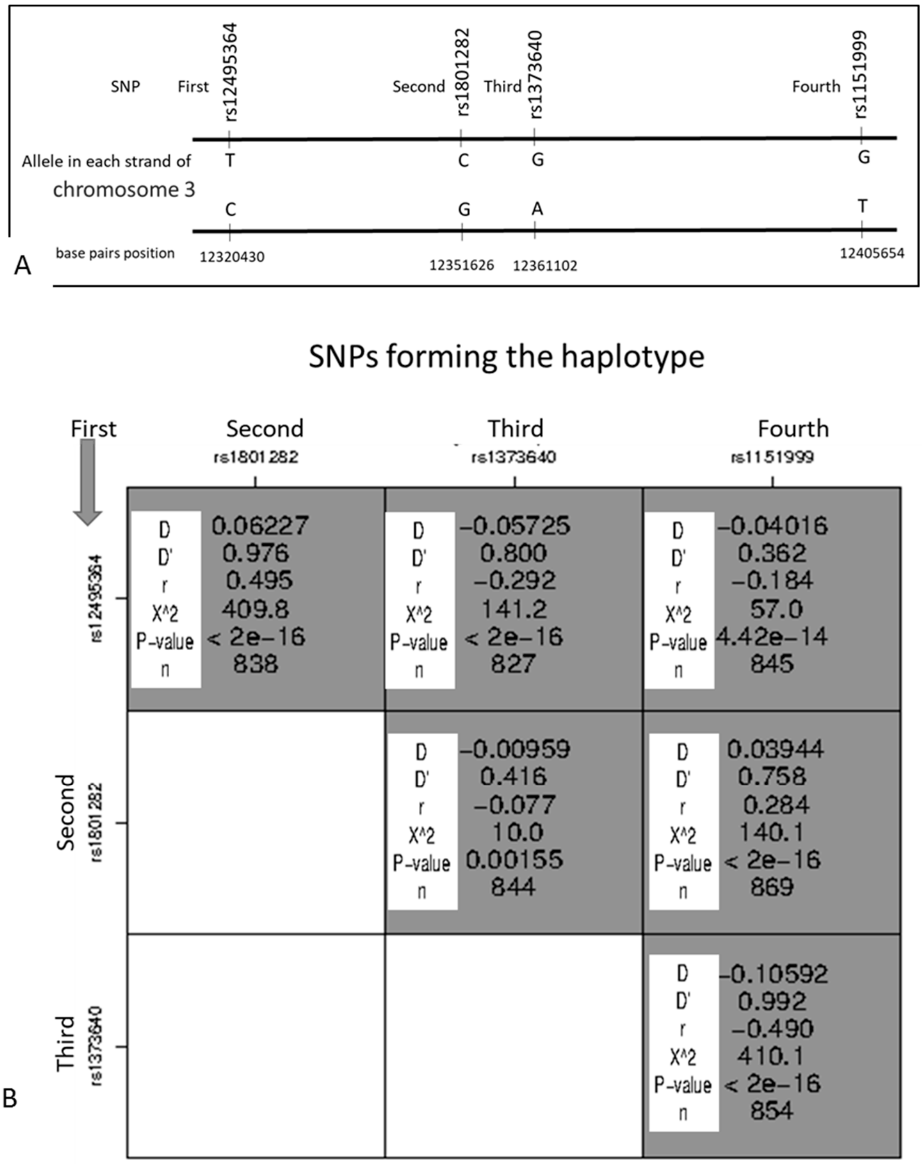

Figure S2. Estimation of the five most frequent haplotypes in different group comparisons, and multiple logistic regression analyses for effects of haplotypes in each diseased phenotype: (A) Healthy versus Periodontitis; (B) Healthy versus P+T2DM; (C) Healthy versus Periodontitis + P+T2DM; (D) Periodontitis versus P+T2DM.

**A - Healthy *versus* Periodontitis**							
**Haplotype association with response (n=695, adjusted by Age+Sex+Smoke)**							
	**rs12495364**	**rs1801282**	**rs1373640**	**rs1151999**	**Freq**	**OR (95% CI)**	**p-value**
1	T	C	G	G	0.3381	1.00	-
2	T	C	A	T	0.2484	1.04 (0.76 - 1.41)	0.81
3	C	C	G	T	0.177	1.25 (0.91 - 1.73)	0.17
4	T	C	G	T	0.1446	1.49 (1.05 - 2.10)	0.025
5	C	G	G	G	0.0747	0.87 (0.54 - 1.39)	0.56
rare	-	-	-	-	0.0173	0.39 (0.12 - 1.21)	0.1
**Global haplotype association p-value**: 0.03							
**B - Healthy *versus* P+T2DM**							
**Haplotype association with response (n=531, adjusted by Age+Sex+Smoke)**							
	**rs12495364**	**rs1801282**	**rs1373640**	**rs1151999**	**Freq**	**OR (95% CI)**	**p-value**
1	T	C	G	G	0.3302	1.00	-
2	T	C	A	T	0.2484	1.12 (0.69 - 1.79)	0.65
3	C	C	G	T	0.1913	2.01 (1.28 - 3.16)	0.0027
4	T	C	G	T	0.1317	1.60 (0.96 - 2.64)	0.07
5	C	G	G	G	0.074	1.06 (0.54 - 2.07)	0.87
rare	-	-	-	-	0.0244	1.24 (0.38 - 4.08)	0.72
**Global haplotype association p-value**: 0.025							
**C - Healthy *versus* Periodontitis + P+T2DM**							
**Haplotype association with response (n=880, adjusted by Age+Sex+Smoke)**							
	**rs12495364**	**rs1801282**	**rs1373640**	**rs1151999**	**Freq**	**OR (95% CI)**	**p-value**
1	T	C	G	G	0.3302	1.00	-
2	T	C	A	T	0.2483	1.09 (0.74 - 1.60)	0.65
3	C	C	G	T	0.1882	1.73 (1.21 - 2.48)	0.0029
4	T	C	G	T	0.1425	1.23 (0.81 - 1.86)	0.33
5	C	G	G	G	0.0723	1.03 (0.59 - 1.81)	0.91
rare	-	-	-	-	0.0184	2.03 (0.71 - 5.85)	0.19
**Global haplotype association p-value**: 0.052							
**D - Periodontitis *versus* P+T2DM**							
**Haplotype association with response (n=534, adjusted by Age+Sex+Smoke)**							
	**rs12495364**	**rs1801282**	**rs1373640**	**rs1151999**	**Freq**	**OR (95% CI)**	**p-value**
1	T	C	G	G	0.3219	1.00	-
2	T	C	A	T	0.2469	1.04 (0.69 - 1.56)	0.86
3	C	C	G	T	0.199	1.44 (0.98 - 2.13)	0.065
4	T	C	G	T	0.1505	0.96 (0.62 - 1.49)	0.85
5	C	G	G	G	0.0673	1.02 (0.56 - 1.87)	0.95
rare	-	-	-	-	0.0145	3.70 (1.00 - 13.66)	0.051
**Global haplotype association p-value**: 0.19							
