# Supplementary material for: Evidence Linking PPARG Genetic Variants with Periodontitis and Type 2 Diabetes Mellitus in a Brazilian Population
Source: Int J Mol Sci. 2023 Apr 5;24(7):6760. doi: 10.3390/ijms24076760 (PMC10095581; doi:10.3390/ijms24076760)
Supplement: Supplementary file 1 [file ijms-24-06760-s001.zip › 3_SupplementaryTable.pdf]

Supplementary material

Evidence linking PPARG genetic variants with periodontitis and type 2 diabetes mellitus in a Brazilian population

Table S1. Information regarding the four genotyped polymorphisms and assays

SNP	Assay ID	Position*, Alleles and type of SNP	Gene	Call rate (%) †
rs12495364	C___9384378_10	chr3:g.12320430, T>C, Intron Variant	*PPARG*	94.55
rs1801282	C___1129864_10	chr3:g.12351626, C>G Missense Variant	*PPARG*	94.97
rs1373640	C___1129853_10	chr3:g.12361102, A>G, Intron Variant	*PPARG*	94.97
rs1151999	C___8756574_10	chr3:g.12405654, G>T, Intron Variant	*PPARG*	96.92

* Genome Reference Consortium Human Build 38 (GRCh38.p12);
†Represents the amount of genotyping performed with success (all subjects), in percentage.

Table S2. Allele and genotype frequencies comparisons between groups

SNP	Healthy n=345	Periodontitis n=349	*p-value*	Healthy n=345	P+T2DM n=202	*p-value*	Periodontitis n=349	P+T2DM n=202	*p-value*
rs12495364 (T>C)									
Alleles									
T	490 (71.0%)	502 (71.9%)		490 (71.0%)	101 (25.0%)		502 (71.9%)	101 (25.0%)	
C	180 (26.1%)	176 (25.2%)	0.71	180 (26.1%)	227 (56.2%)	0.19	176 (25.2%)	227 (56.2%)	0.11
MAF	0.27	0.26		0.27	0.31		0.26	0.31	
Genotypes									
TT	178 (51.6%)	193 (55.3%)		178 (51.6%)	81 (40.1%)		193 (55.3%)	81 (40.1%)	
TC	134 (38.8%)	116 (3.3%)		134 (38.8%)	65 (32.2%)		116 (3.3%)	65 (32.2%)	
CC	23 (6.7%)	30 (8.6%)	0.24	23 (6.7%)	18 (8.9%)	0.28	30 (8.6%)	18 (8.9%)	0.27
H-W Equilibrium	0.89	0.05		0.89	0.48		0.05	0.48	
rs1801282 (C>G)									
Alleles									
C	636 (92.2%)	611 (87.5%)		636 (92.2%)	356 (88.1%)		611 (87.5%)	356 (88.1%)	
G	54 (7.8%)	67 (9.6%)	0.18	54 (7.8%)	32 (7.9%)	0.39	67 (9.6%)	32 (7.9%)	0.80
MAF	0.09	0.08		0.09	0.08		0.08	0.08	
Genotypes									
CC	276 (80.0%)	293(83.9%)		276 (80.0%)	174 (8.6%)		293(83.9%)	174 (8.6%)	
CG	59 (17.1%)	50 (14.3%)		59 (17.1%)	145 (71.8%)		50 (14.3%)	145 (71.8%)	
GG	4 (1.9%)	2 (0.6%)	NA	4 (1.9%)	16 (7.9%)	0.47	2 (0.6%)	16 (7.9%)	NA
H-W Equilibrium	0.55	1.00		0.55	**0.04**		1.00	**0.04**	
rs1373640 (A>G)									
Alleles									
G	293 (42.5%)	303 (43.4%)		293 (42.5%)	253 (62.6%)		303 (43.4%)	253 (62.6%)	
A	313 (45.4%)	361 (51.7%)	0.66	313 (45.4%)	99 (24.5%)	0.55	361 (51.7%)	99 (24.5%)	0.34
MAF	0.26	0.26		0.26	0.28		0.26	0.28	
Genotypes									
GG	174 (50.4%)	189 (54.1%)		174 (50.4%)	90 (44.5%)		189 (54.1%)	90 (44.5%)	
GA	145 (42.0%)	128 (36.6%)		145 (42.0%)	73 (36.1%)		128 (36.6%)	73 (36.1%)	
AA	16 (4.6%)	22 (6.3%)	0.27	16 (4.6%)	13 (6.4%)	0.47	22 (6.3%)	13 (6.4%)	0.60
H-W Equilibrium	0.05	1.00		0.05	0.71		1.00	0.71	
rs1151999 (G>T)									
Alleles									
T	389 (56.4%)	415 (59.6%)		389 (56.4%)	234 (64.6%)		415 (59.6%)	234 (64.6%)	
G	301 (43.6%)	281 (40.4%)	0.22	301 (43.6%)	128 (35.4%)	**0.0096**	281 (40.4%)	128 (35.4%)	0.11
MAF	0.43	0.40		0.43	0.36		0.40	0.36	
Genotype									
TT	109 (31.6%)	128 (36.8%)		109 (31.6%)	74 (40.9%)		128 (36.8%)	74 (40.9%)	
TG	171 (49.6%)	159 (45.7%)		171 (49.6%)	86 (47.5%)		159 (45.7%)	86 (47.5%)	
GG	65 (18.8%)	61 (17.5%)	0.35	65 (18.8%)	21 (11.6%)	**0.032**	61 (17.5%)	21 (11.6%)	0.19
H-W Equilibrium	0.91	0.37		0.91	0.75		0.37	0.75	0.35

P+T2DM = Periodontitis+Type 2 Diabetes Mellitus. NA= Not analyzed. MAF= Minor Allele Frequencies. H-W Equilibrium = Hardy Weinberg Equilibrium; **Bold font** indicates p < 0.05. p-value evaluated by the chi-square test.
